# Causal Evidence and Dispositions in Medicine and Public Health

**DOI:** 10.3390/ijerph17061813

**Published:** 2020-03-11

**Authors:** Elena Rocca, Rani Lill Anjum

**Affiliations:** Norwegian University of Life Sciences, Centre for Applied Philosophy of Science, 1430 Ås, Norway; rani.anjum@nmbu.no

**Keywords:** causality, public health, medicine, pharmacovigilance, predictions, health outcomes, research methods, dispositions, evidential pluralism

## Abstract

Since the introduction of evidence-based medicine, there have been discussions about the epistemic primacy of randomised controlled trials (RCTs) for establishing causality in medicine and public health. A growing movement within philosophy of science calls instead for evidential pluralism: that we need more than one single method to investigate health outcomes. How should such evidential pluralism look in practice? How useful are the various methods available for causal inquiry? Further, how should different types of causal evidence be evaluated? This paper proposes a constructive answer and introduces a framework aimed at supporting scientists in developing appropriate methodological approaches for exploring causality. We start from the philosophical tradition that highlights intrinsic properties (dispositions, causal powers or capacities) as essential features of causality. This abstract idea has wide methodological implications. The paper explains how different methods, such as lab experiments, case studies, N-of-1 trials, case control studies, cohort studies, RCTs and patient narratives, all have some strengths and some limitations for picking out intrinsic causal properties. We explain why considering philosophy of causality is crucial for evaluating causality in the health sciences. In our proposal, we combine the various methods in a temporal process, which could then take us from an observed phenomenon (e.g., a correlation) to a causal hypothesis and, finally, to improved theoretical knowledge.

## 1. Introduction

The current evidence-based paradigm requires that the most reliable scientific evidence is used to increase patient safety by predicting how a certain intervention might affect health and well-being. This is true both in a strictly clinical context, where doctors and patients need to evaluate the potentiality of interventions for the single case, and in public health, where policy interventions are needed at the population level. How should such evidence best be generated? This is an ongoing debate in medicine and public health, but also in philosophy of science.

Recently, there has been some discussions about the epistemic primacy of statistical approaches, such as randomised controlled trials (RCTs), for establishing causality and predict the health outcomes of medical interventions. In the recent article ‘Understanding and Misunderstanding Randomized Controlled Trials’, for instance, Deaton and Cartwright summarise a number of arguments against the dominant reliance on RCTs over every other form of causal evidence [1]. This article has received considerable attention, partially because it is written within a growing movement that calls for evidential pluralism in the medical and health sciences [2,3,4,5,6,7]. The main idea is that evidence from RCTs can provide useful causal information, but only when combined with evidence from other methods for causal inquiry. The purpose of causal evidence would then be to develop the theoretical knowledge and to advance the understanding of ‘the hows and whys’ underlying the statistical correlations.

Scholars who argue that we need plural methods to establish causality use different types of arguments and have different emphasis. Some traditions, such as critical realism, are primarily critical of the ontological bias in scientific methodology; they argue that standard ways to evaluate causal evidence mistakenly rely on a positivist, Humean conception of the nature of causality [2,8,9]. Others are more concerned with epistemological issues of how we can know about causality using different methods, without considering the ontological question of what causality is [5,10]. However, the ontological and epistemological traditions generally agree that what counts as ‘the best causal evidence’ differs depending on the research question and the research context. This stance is in opposition to a formal, probabilistic tradition which is primarily interested in the mathematical relationship between a certain hypothesis and a certain evidence, without considering the specific causal content or explanation to the evidence itself [11]. 

Although the formal approach is still dominant, there is increasing awareness among scientists that the evaluation of causal hypotheses cannot be restricted to purely statistical standards [12,13]. In a recent commentary in *BMJ Evidence Based Medicine*, a large group of clinicians, medical researchers and philosophers made a common appeal to evidence-based medicine to expand its notion of ‘evidence’:

The rapid dominance of evidence based medicine has sparked a philosophical debate concerning the concept of evidence… The undersigned include 42 clinicians and philosophers from interdisciplinary research networks working specifically on questions related to causality in medicine worldwide. Our research has developed out of a conviction that philosophical analysis ought to have a direct impact on the practice of medicine. In particular, if we are to understand what is meant by ‘evidence’, what is the ‘best available evidence’ and how to apply it in the context of medicine, we need to tackle the problem of causality head on… Establishing causality often requires the use of multiple methods, since no single method will be universally applicable or perfect for this purpose

[14] (p. 1)

How should this call for evidential pluralism be understood? How useful are the various methods available for causal inquiry? Further, how should different types of causal evidence be evaluated? Among philosophers of science, various answers to this question have been proposed. For instance, Russo and Williamson (2007) state that a causal claim must be supported both by evidence of difference-making from population studies and from evidence of the underlying mechanism, and that each of these approaches compensate for the weakness of the other [15]. Others are trying to expand formal approaches to the inclusion of different types of methods and the amalgamation of different types of evidence [16,17].

In this paper, we propose our own version of evidential pluralism. This version is based on the philosophical idea that any type of scientific claim, including causal claims within medicine and public health, should seek to say something about a system’s intrinsic properties, as well as their mutual influence and causal interaction. Our aim here is to introduce a framework to support scientists in developing appropriate methodological approaches for exploring causality. This is relevant for ensuring that all the evidence necessary for the safe treatment of individuals and populations is considered.

## 2. Dispositions and Science

In her discussion about causal evidence and scientific methods, Cartwright urges that the whole scientific enterprise is about finding properties, or potentialities, which she calls *stable capacities* [18]. For instance, an RCT might show that, for a certain sample of population, aspirin relieves headache in more instances than a sugar pill does. This observation is interesting insofar as it allows us to make a claim about a capacity of aspirin, which is generated from its properties. Such capacity, we can say, then works as the truth-maker of causal claims: ‘aspirin has a capacity to (causally) relieve headache’.

What Cartwright calls ‘capacities’ are also commonly referred to as *causal powers*, or *dispositions*, with a very similar meaning [19,20]. Despite some disagreements over philosophical details, proponents of capacities, powers and dispositions generally agree that these are properties or potentials of things or systems, that can become manifested under certain conditions. The philosophy of dispositions descends from an ancient tradition going back to Aristotle, but which has had a revival in recent decades. Indeed, in philosophy of science, there has been a tendency to shift the attention away from ideal and extrinsic laws of nature onto the intrinsic causal powers of things and systems [18,21]. Shifting the attention to the dispositions, or properties, of a system corresponds to looking at the causal mechanism, the interactions and the dynamics at place. It is no longer sufficient to notice a lawlike regularity in nature, for instance, that holds under some ideal, normal or similar conditions. Rather, one now wants to understand the intrinsic dispositions and causal capacities of the system that allow such regular behaviour [20]. In other words, one is now seeking a deep causal understanding about *why* and *how* something might or might not happen. This contrasts with the investigation into *whether* and *how often* something happens, which is typically tested via statistical significance, relative frequencies, and so on [22].

There is a flourishing literature about the metaphysics of dispositions and powers (recent edited volumes include [9,23,24,25,26]). The question here is whether thinking in terms of dispositions can help the discussion about causal evidencing in medicine and public health, and if so, how? If we acknowledge that the health sciences should be concerned with understanding the underlying causal powers, capacities or dispositions of observable processes and events, then what are the specific advantages and disadvantages of different types of scientific evidence for this purpose? There has been comparative little discussion on these topics, at least to our knowledge.

In what follows, we will address these questions in more detail. As the theoretical framework for our discussion, we adopt a particular idea within the literature on dispositions: the theory of causal dispositionalism, developed by Anjum and Mumford over the last decade (for a detailed overview of the theory, see [27]). The aim is to make the methodological implications of this specific philosophy of causality more explicit and show the relevance of considering philosophy of causality for medicine and public health.

## 3. Dispositionalism about Causality

In the Anjum–Mumford dispositionalist theory, causes come from dispositions or ‘causal powers’. What is a disposition in this specific view, and how exactly does it relate to causality?

A disposition is, in this view, an *intrinsic* property, belonging to some particular thing, individual or process. That dispositions are intrinsic in this way is crucial for causality in medicine. For instance, an intervention cannot be said to cause an effect unless it has an intrinsic disposition toward the recovery. Dispositional properties can exist unmanifested. A woman can be fertile without ever becoming pregnant, but once fertility is manifested in pregnancy, causality has happened. From this philosophical perspective, causality requires more than a single disposition. In order to manifest its effect, the disposition must interact with a number of other dispositions, or ‘mutual manifestation partners’ [28]. These are reciprocal partners for the manifestation, such as the dispositions of the sperm, ovum, uterus, placenta, and so on, to initiate and maintain the pregnancy [29].

All dispositions will also have a degree of tendency, which can range from very weak to very strong. Something can be more or less robust, soluble, toxic, vulnerable, and so on. The workings of intrinsic properties could for example give a statistically weak tendencies, such as the tendency of oral contraception to produce thrombosis. But in some individuals, this disposition might be very strong. Still, no matter how strong the tendency is, a disposition cannot manifest alone or in isolation. Causal production is then, in this view, always a matter of complex interaction of multiple dispositions, many of which are represented by the background conditions. For instance, a patient must be an appropriate manifestation partner for the medical intervention to work, or else be a non-responder. But rather than seeing the patient as the background condition for the intervention to do its causal work, which is the standard view of causality, causal dispositionalism takes all contextual factors that influence the outcome as causes themselves. For instance, the outcome of behaviour-changing techniques in improving lifestyle of individuals will depend not only on the type of technique and the single treated individual, but also on what is already there in the person’s context, such as her current life phase, health, occupation, social status and family situation. A cancer survivor might be more disposed to lifestyle changes and thus a better mutual manifestation partner for the intervention than someone with a good general health or low motivation. Individuals, and even whole populations, can be thought of as mutual manifestation partners for an intervention to do its causal work—for instance, when a social intervention is tested.

Because the effect is a result of the complex interaction of different dispositions, the causal process will also be highly sensitive to context, according to this theory. What a disposition does in one context, together with a certain combination of mutual manifestation partners, will be different from what it does in another combination. For a causal dispositionalist, therefore, the default expectation should be that the same disposition will tend to produce different effects depending on which dispositions it interacts with. A biomedical example is the way in which thalidomide tends to cause teratogenicity, but only if taken when the embryo is in the first trimester in a pregnant woman [30]. A famous public health example is given by Cartwright, showing that the intervention of teaching nutrition to mothers tends to cause an improvement in the children’s health. This did not however work in communities where the main person responsible for planning and preparing the family meals was not the mother, but rather the mother-in-law [31].

To sum up, causal dispositionalism defines causality as the result of the interaction of multiple intrinsic properties, or dispositions.

## 4. Detecting Dispositions

If we understand causality dispositionally, as briefly described here, then how should this affect the way we study, establish, and evaluate causality in medicine? In dispositionalist terms, to establish a causal link between an intervention *I* and an effect *E* corresponds to establishing whether *I* has intrinsic dispositions that, in combination with other dispositions, can eventually produce *E*. Different types of methods will have some strengths and some limitations for the purpose of finding and establishing intrinsic dispositions, as we now go on to show. The methods discussed are listed in Table 1.

Note that this is not meant as an exhaustive list of methods, but as representative enough to demonstrate that the dispositionalist theory suggests that we need more than one type of method to establish causality. Further, the purpose of this discussion is not to set some quality standards for the individual methods, but rather to offer a more general framework to show how the various methods can complement and inform each other in the causal inquiry. This paper is then presenting an ontologically informed argument in support of methodological pluralism, motivated by a philosophical theory of what causality is, rather than a purely epistemological point about how we can establish causality empirically [2].

### 4.1. Experimentation with Lab Models

Lab experimentation is commonly used to investigate the causal mechanisms underlying correlations, clinical reports and various other types of observations. This is done in order to evaluate the biological plausibility of a causal claim. For instance, if a certain genetic mutation is correlated with increased vulnerability to a class of chemicals, one can investigate whether there is a biological explanation for such correlation by setting up experiments with lab models. This includes a large variety of techniques and models: in vivo, in vitro and ex vivo experimentation with animals, cell culture, microscopy, genetic manipulation and post-mortem examinations, to name only some. 

Experimentation with lab models has certain strengths for establishing dispositions. Specifically, they allow us to study the causal processes in detail and over time, which can give insights into what a particular disposition is and how it interacts with different manifestation partners and under a variety of conditions and contexts.

On the other hand, lab experiments have some epistemic limitations from the dispositionalist perspective. Since a disposition will produce different effects in combination with different sets of other dispositions, we should not expect that an intervention will produce exactly the same effect under some controlled lab conditions as they do in the clinic. However, these contextual differences can be harnessed in an attempt to understand more about a disposition and its various potential manifestation partners for a particular effect. Again, this requires a reciprocal collaboration between basic research and the clinic [22,32].

Some examples will help with illustrating these advantages and disadvantages for establishing dispositions. In colorectal cancer, the receptor for epidermal growth factor (EGFR) is highly expressed [33]. In lab experiments, the monoclonal antibody cetuximab was shown to bind EGFR with high affinity, which suggested that cetuximab might have a disposition to slow down the progress of colorectal cancer (by blocking EGFR) [34]. However, this disposition does not manifest in the clinic in patients with a mutation in the KRAS protein [35]. This indicated that KRAS is a necessary mutual manifestation partner for cetuximab to do its causal work, which again contributed to expand our causal knowledge of the molecular mechanisms by which cetuximab blocks the molecular pathways downstream of EGFR. Once we know why the effect did not happen without this disposition of the KRAS protein, this different set of dispositions can be modelled in the lab—for instance, using cells expressing mutated KRAS.

The dispositional strength of lab experimentation is not restricted to the biological realm, but it can sometimes expand to causal mechanisms of a social type. One example is the investigation of the mechanisms underlying addiction. Lab animals helped clarify that social isolation, especially during early life stages, is a factor that can contribute to increased addictive behaviour in adult life [36]. 

### 4.2. Patient Narrative

Narrative-based medicine was defined as ‘a fundamental tool to acquire, comprehend and integrate the different points of view of all the participants having a role in the illness experience’ [37]. The attention to patient narrative as an essential tool for medicine has been increasingly emphasised in the last 30 years [38]. The main message of this movement is that medical care should shift its primary focus away from problem solving and toward understanding, but that this can only be done by considering patient’s stories. 

One thing is that listening to patient narratives is considered essential to understand their needs and values [39]. Another point, more relevant for causal inquiry, is that patients can provide essential elements of the causal story, through the subjective narration of their experience [40,41]. By listening to the story that a patient tells, and to the way in which she chooses to tell it, one can map detailed contextual information that could contribute to explaining the patient’s current condition. How does this work? Let us illustrate this with an example from general practice.

A patient with a long history of unsolved chronic muscle cramps, symptoms of epilepsy, eating disorders, anxiety attacks and self-harm, was further harmed by adverse effects from long-term medical treatments and surgery. For decades, medical examinations could not uncover the aetiology of her complex condition, until analysis of her narrative, previously overlooked, indicated an important role of childhood trauma, violence and abuse [42]. This type of patient-based evidence has been key to acknowledging cumulative childhood stress as a plausibly strong disposition toward immune dysfunction and other chronic conditions in adulthood [43,44,45,46]. Because of this and many similar stories, there is a growing interest in the way that patient experiences can serve as causal evidence and in how this evidence should be evaluated. 

We see some clear dispositional strength in considering evidence from patient narratives. Rich patient information allows the detection of relevant dispositions that are uniquely combined in this patient. This could be information of a biomedical or biographical nature or concerning lifestyle or life situation. By considering these, one might get a better idea of the causal story leading up to their condition, as well as causally relevant information about what might be the best treatment for this particular individual.

On the other hand, there are some limitations from the dispositionalist perspective. It is not easy to establish which of the many elements in the narrative that represent relevant dispositions for the illness or treatment. Indeed, if one does not have additional theoretical knowledge about what factors could influence the condition or treatment in general, one might not even know what to notice in the narrative as potentially informative. This is a problem for all the methods based on in-depth analyses of one or a few instances.

An example can be found in the practice of pharmacovigilance: the post-marketing monitoring of secondary effects of drugs. The aim of pharmacovigilance is to build a hypothesis of causality between a drug and a symptom, based on spontaneous reports of patients’ experiences. The WHO Collaborating Centre for International Drug Monitoring, Uppsala Monitoring Centre, has the world’s largest database of spontaneous reports of suspected adverse drug reactions. They use this database to assess whether the reported reactions were in fact caused by an intrinsic disposition of the drug. Although pharmacovigilance is largely based on statistical analysis of such databases, a key step in the generation of a causal hypotheses is to assess the likelihood of a causal relationship based on a single or a few patient reports. In the words of pharmacovigilance expert, Rebecca Chandler, ‘*it is logical that clinical stories contained in adverse event reports […] are integral to the development of hypotheses of drug safety concerns*’ [47] (p. 23). In order to catch which of the details in the patient stories point to relevant causal dispositions, however, the clinical narration must be read through the lenses of some kind of theoretical knowledge. This could, for instance, be knowledge about the drug’s components, their mechanism of action, side effects of similar drugs or pathological mechanisms underlying the reported symptom.

The practice of pharmacovigilance is thus an excellent example of how using databases of patient narratives, in combination with theoretical knowledge and clinical expertise, can be effective for detecting causal dispositions.

### 4.3. Case Studies

A case study is the detailed description and analysis of one single instance of a certain type of phenomenon. In medicine, it typically consists of the analysis of one medical case that is of particular interest—for instance, if it is new, rare and unusual, or somehow contributes to the advancement of medical understanding. For the purpose of public health, case studies of a particular context are often used to gain insights into the mechanisms underlying observed correlations. For instance, in order to understand the causal relationship between loneliness, social isolation and general health in older adults, case studies were conducted in specific contexts [48].

In medicine and public health, there is disagreement about the epistemic role of such a type of study. One view, which is dominant in the evidence-based paradigm, is that a case study provides qualitative knowledge only about the analysed context and can therefore not be used to derive general causal claims. In contrast, others argue that a detailed case study might have the potential for generalisation across similar instances. According to this view, depending on the research question, generalising from the detailed understanding of one instance can be equally justified (or equally wrong) as generalising from the statistical analysis of a few parameters in a larger population [49,50]. This latter point is in line with dispositionalism: since dispositions are intrinsic properties, they tell us something general about the potentiality of a thing or a system, even if they are rarely manifested. Accordingly, if one knows how the disposition manifests (in which contexts and in combination with which manifestation partners), this knowledge can potentially be applied beyond the single case.

For the purpose of uncovering intrinsic dispositions, case studies are important tools. Indeed, they typically include a variety of scientific methodologies to gain a full overview of the context under scrutiny. A well-conducted case study can converge medical examinations, lab analyses, medical history, qualitative interviews, analysis of social context and of lived experiences, and can therefore be analysed from the perspectives of different experts. This detailed and complex analysis aims to *understand* how the dispositions at place interact with each other. Moreover, it allows the description of rare or unexpected effects in a particular individual or context, suggesting previously unknown dispositions or mutual manifestation partners. This in turn can contribute to theory development of causal mechanisms. 

On the other hand, case studies have some limitations for the purpose of detecting dispositions. Specifically, the information in the case study is already defined, filtered and interpreted, restricting the causal information gathered from the instance analysed. This is especially relevant in medicine, where case studies translate the narrative and perspective of the patient into medical terms and categories. In this process, the researcher filters the information that is considered relevant, in light of their knowledge, interest and research question. When we miss the plurality of perspectives, we might also miss important information about the dispositions at place in the single case.

This can be exemplified with a case study in the field of medical anthropology, aimed at investigating the causal relationship between the high incidence of illness in a low income African-American neighbourhood and pollution from surrounding industries [51]. With this deep analysis, the author not only draws a general conclusion about the causal relationship between the race and class experience and the susceptibility to environmental risk. She also points out that, without a deep understanding of the single case, we risk missing and misinterpreting crucial causal evidence:

…*in collecting surface soil samples, testers had actually sampled new dirt that residents had imported and put over their old, contaminated dirt… ‘They sent out some people to do that testing out here and they scooped a little bit of dirt with spoons on the ground. Hey, I done put dirt on top of dirt trying to get rid of the floods and things we been having out here for years’*.

[51] (p. 117)

There are, however, also case studies that are more biomedical in nature and that can potentially be of great importance for discovering new and unexpected dispositions. Consider, for instance, the infamous case reports of malformations after embryonic exposure to the drug thalidomide. The study of embryonic malformation in cases of maternal exposure to thalidomide suggested that certain tissues of the developing embryo were, contrary to previous assumptions, manifestation partners for the drug. These reports led to investigations that ultimately resulted in new theories of teratogenicity, safe placental barrier, and sensitivity in different species. This in turn motivated mechanistic studies, uncovering, for instance, the disposition of thalidomide to inhibit angiogenesis and, consequently, to treat myeloma [52].

### 4.4. Case Control and other Retrospective Studies

Case control and retrospective studies are commonly used in medicine and epidemiology. In case control studies, one considers a group of people with a similar condition and tries to find a factor which is common to all the participants’ medical history, but absent in a control group. This means that one is looking for a common cause of social, medical or toxicological type, that might explain the difference in outcome between the test group and the control.

The dispositionalist advantage of this study design is that, while looking for a factor that supposedly made a difference between two samples of the same population, it allows a detailed causal study of small samples, such as outlier cases or very rare conditions. In this way, one might detect dispositions and manifestations that are otherwise too rare to show up through other statistical approaches. Such studies also acknowledge the temporal aspect of causality—that dispositions might take a long time to manifest as a detectable outcome. This could in turn contribute to discover a causal mechanism.

The downside, on the other hand, is that these studies do not specifically look for manifestation partners that might contribute to the outcome but are not common to all the participants. Indeed, they are designed to find common dispositions across different contexts. We also need to point out that the output of these studies are correlations and not dispositions. Without a further theory of mechanism that can plausibly account for how the common factor brings about the common outcome, we cannot claim to have found an intrinsic property.

This point is not only valid for this experimental design, but for all controlled experiments that are primarily designed for detecting correlations between variables at a statistical level. There is, for instance, a correlation between the exposure to paracetamol and incidence of asthma in children [53]. This provides nothing more than a suggestion that paracetamol disposes toward asthma. The correlation could be due to the fact that exposure to paracetamol and asthma have a common cause—for instance, chronic airway inflammation and infections. As others have already noted [1], such risk of confounding can be reduced with certain experimental designs, but never eliminated. Even a correlation produced by the most sophisticated population trial provides an indication, but never the ultimate evidence, for an intrinsic disposition.

An example of the discovery of a causal disposition from a case control study is the following. Between 1966 and 1969 at the Vincent Memorial Hospital, Massachusetts, eight young women were found to have a rare form of vaginal adenocarcinoma. This observation triggered a retrospective comparative study, which showed that seven of the patients had been exposed to the exogenous hormone stilbestrol during embryonic life [54]. This suggested that the drug disposed toward an anomaly of the vaginal epithelium, which in time could result in cancer. The evidence for this disposition was then strengthened with lab experimentation, and provided new knowledge about the mechanism by which the drug affects the development of vaginal epithelium [55].

### 4.5. Cohort Studies and other Prospective Studies

In cohort studies, a large group of people is observed over a period of time, in which relevant medical events and exposures of every participant are recorded in databases. Such data are then used to answer different research questions about existing correlations between possible risk factors or susceptibility factors and health outcomes, at the population level.

Like retrospective studies, cohort studies also acknowledge the temporal aspect of causality, which is central if one thinks in terms of intrinsic dispositions and their manifestations. One could argue that, since prospective studies record events as they happen over time, rather than being based on memories and existing records, such studies account better for the temporal dimension than retrospective studies. It is, however, not easy to determine how much time an unknown disposition will take to manifest. In order to make a good estimate of this, one must have a great deal of background knowledge and mechanistic understanding of the processes in place, so that the study is not discontinued before it is possible to make the relevant observations. Some dispositions might take generations to develop, as was the case with stilbestrol and vaginal cancer, while others can manifest within seconds.

In addition to the temporal dimension, another dispositional advantage of cohort studies is that they have the potential to address causal complexity and the interactions of different manifestation partners. By broadly monitoring development in a cohort over a long time, one could detect the interdependency of a certain health outcome with numerous factors. In principle, these observations could allow the study of causal complexity by pointing to intrinsic dispositions (e.g., genetics), extrinsic dispositions (e.g., environmental stressors) or even their mutual interactions. Again, since these observations are made at the population level, they just indicate causal complexity, but provide per se no understanding of it: of how and why intrinsic properties interact and manifest. Still, in combination with other approaches, such as deep contextual analyses of single cases, the evidence produced in cohort studies can potentially be a good starting-point for the detection of dispositions.

The dispositionalist downside of this experimental design is that when resources are limited, priority tends to be given to large numbers of people over large numbers of factors monitored. This takes away a great potential of this study design for uncovering causal complexity of dispositions. Normally, research on databases from cohort studies tends to point to the interrelation of a few rather than many factors, normally just two or three.

Cohort studies can be used to discover both biomedical and psychosocial dispositions. For instance, some cohort studies found a positive association between an increased consumption of processed meat and an increased incidence of colorectal cancer [56]. However, this evidence is not conclusive and is only suggestive of an intrinsic disposition of processed meat to contribute to cancer development until further evidence is available. For instance, experimental studies in animals might indicate dose relatedness, reversibility or the biological mechanisms underlying this correlation [57]. In turn, results from animal studies can inform further clinical studies.

Another cohort study is the Norwegian Trøndelag Health study (HUNT), which so far has had over 125,000 participants since 1984 (https://www.ntnu.edu/hunt). In this case, a large range of factors were monitored, with an initial aim to study risk factors of cardiovascular conditions, metabolic conditions and quality of life. Because of this unusually broad approach, researchers have been able to use the study database to uncover a considerable correlation between self-reported childhood trauma and adult comorbidity [46]. That this correlation points to a disposition is supported by mechanistic evidence of how allostatic load can lead to systemic chronic inflammation [58]. Other supporting evidence comes from qualitative, phenomenological studies using patient narratives [42].

### 4.6. Randomised Controlled Trials (RCTs)

The purpose of a randomised controlled trial is to compare the outcome of one or more randomly assigned clinical intervention(s). This is considered the gold standard for causal evidencing in medicine and evidence-based decision making. The reason for this is that it is specifically designed to look for difference makers, while controlling for confounding factors. This means that any difference in outcome between the test group and the control group should be caused by the tested interventions, since all other differences should be homogenously distributed between the two groups. The exclusive reliance on RCTs has been critically debated in recent decades, both from philosophy of science and from the research community (see, for instance, the special issue of *Social Science and Medicine* ‘Randomized Controlled Trials and Evidence-based Policy: A Multidisciplinary Dialogue’, August 2018). Here, we only consider the strengths and limitations of RCTs for establishing intrinsic dispositions and how they interact with other mutual manifestation partners.

An obvious strength of RCTs from the perspective of causal dispositionalism is that dispositions tend to make a difference to the outcome [2]. This means that comparative methods, and particularly RCTs, are best suited for picking out this feature of dispositions. Statistically significant results from an RCT could indicate that the intervention played a causal role for the outcome, either as an intrinsic disposition or as a necessary background condition (a sine qua non). The latter would be where something that was necessary for the effect, although it did not as such cause the effect. Hypothetically speaking, if one had no understanding of the underlying biological mechanisms, one might, for instance, find that hysterectomy significantly reduces the risk of unwanted pregnancy and take this to mean that the uterus is the cause rather than a necessary condition for pregnancy. This shows how what is the advantage of RCTs from a dispositionalist perspective is also the reason why they cannot produce dispositional evidence on their own.

Moreover, RCTs are less suited for identifying mutual manifestation partners for a disposition, since the focus is on one or a few particular interventions for which there can be a control or comparison, and on one or a few outcomes. The experimental design is intended to minimise complexity—for instance, through strict inclusion and exclusion criteria [2,59,60]. From a dispositionalist perspective, heterogeneity of the outcome could point to important interactions and manifestation partners [22]. This type of causal knowledge must thus be found via other methods, such as lab experiments, case control studies, or observational studies.

An example can illustrate this. Large randomised controlled trials showed a convincing association between the use of statins as a pharmacological treatment of hypercholesterolemia and a lower incidence of cardiovascular mortality [61]. These studies, combined with the known mechanism by which statins interfere with cholesterol biosynthesis and thereby with lipid metabolism, provided good evidence that statins have an intrinsic disposition to prevent heart disease by lowering the concentrations of lipids in the blood. However, the response to this type of therapy was highly heterogeneous [62], which points to a number of unknown manifestation partners, and unknown dispositions of the drug. Understanding such complexity requires a different type of evidence—for instance, pharmacogenetics observational studies analysing the genetic underpinning to the heterogeneous drug response [63].

### 4.7. N-of-1 Trials

In an N-of-1 clinical trial, several different interventions are tested in random sequence in the same patient or context, and the outcomes are compared. The aim of this type of experiment is to find which treatment is correlated to the best outcome in the single case, especially for complex, context-sensitive and multi-factorial treatments and conditions. For instance, the correlation between type of diet and symptoms of an illness can be tested with N-of-1 trials, with the advantage that the patient works as her own control. Having a perfectly matching control is important, since the response to a dietary change is interrelated to a number of other known and unknown individual variables.

N-of-1 trials are good tools for establishing difference making in a unique context. Difference making, we said, can be a good indicator of dispositions, since dispositions tend to make a difference. This is because of their comparative design and because repeatable interventions can be randomly allocated, sometimes even blindly. N-of-1 trials therefore have some epistemic advantages for establishing dispositions, especially because they allow us to monitor the effects of different interventions within the same patient. This means that these studies can help reveal how different dispositions manifest in interaction with a particular patient and their unique combination of dispositions, even if this specific combination of mutual manifestation partners is never repeated in another patient.

There are, however, also some epistemic limitations. Further, this type of experiment, taken in isolation, only establishes a correlation. To move from this to claiming that the correlation is an expression of an intrinsic disposition, we will need additional theoretical knowledge and high quality patient-based evidence.

An N-of-1 trial could, for instance, be used in cases where we seek to understand which factors could influence the intensity and frequency of symptoms, such as migraine attacks, epileptic seizures, cramps or episodes of memory loss. Although different people might share the same disposition toward migraine, the triggers and the vulnerability to these will typically vary from one individual to another. Such individual variation, which is essential in causal dispositionalism, is not a problem in these trials, since they already consider the most relevant ‘subpopulation’. Still, any such individual outcomes will remain correlations until a further plausible mechanism of action is provided.

For example, N-of-1 trials were used in single patients with osteoarthritis to compare the effectiveness of paracetamol and ibuprofen to tailor treatment to the individual patient [64]. While this was useful for choosing the best treatment (the correct manifestation partner) for the patient’s unique set of dispositions, patients were also encouraged to monitor other factors that, in interaction with the drugs, seemed to affect the health outcome (eating habits, etc.). This is valuable for clarifying whether the therapeutic disposition belongs to the drug or to some other contextual factor, or both.

## 5. Combining Evidence for Establishing Dispositions

We now summarise the advantages and disadvantages of the reviewed research methods for the purpose of detecting dispositions and manifestation partners, as well as indicating the corresponding recommendations, in the following table (Table 2). 

**Figure 1:**
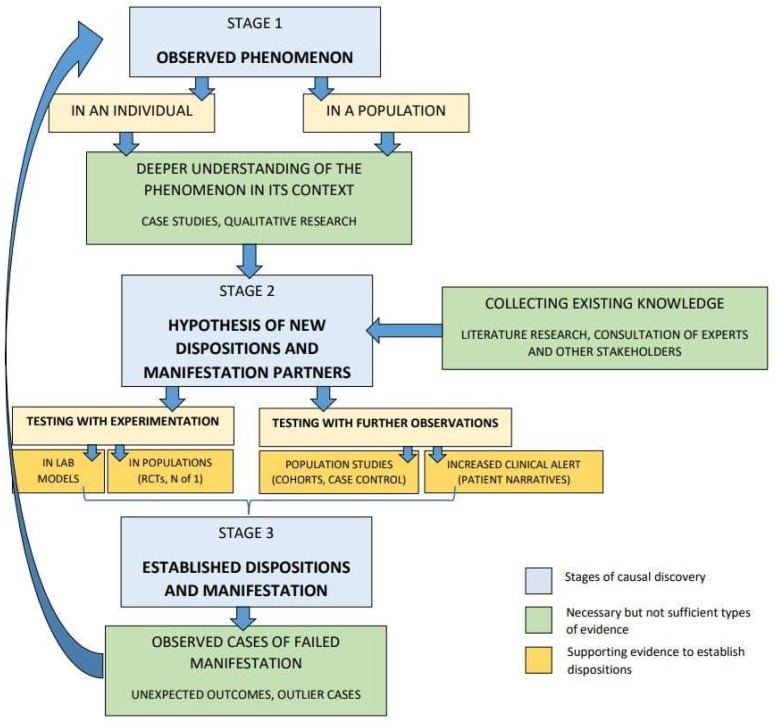
A dispositionalist approach to methodological pluralism for causal evidencing in medicine and public health.

Looking at the strengths and limitations of each of these methods, we can see that it is necessary to combine them for the purpose of causal enquiry and evidencing. That said, our analysis also shows why the different methods would have to play different roles at different stages in this process. Specifically, we have seen that, while all types of evidence contribute to detect dispositions and their manifestation partners, some types of evidence play an indispensable role for establishing their intrinsicality. This allows us to support a pluralist methodology while at the same time offer a way to prioritise which methods to use at different stages. In light of the dispositionalist theory, as outlined above, we thus propose the following approach to causal inquiry and evidencing in medicine and public health (Figure 1):

In Figure 1, we identify three stages of the causal discovery process (blue boxes): (i) observed phenomenon; (ii) hypothesis for new dispositions and manifestation partners; (iii) established dispositions and manifestation partners. In order to proceed from one stage to the next, certain types of evidence are necessary but not sufficient in every type of causal inquiry (all the green boxes). Among all types of causal evidence, these are the most useful for establishing intrinsicality. Moreover, some additional supporting evidence is still required to establish a disposition (some but not necessarily all of the yellow boxes). Different types of supporting evidence can be more suitable in different cases, depending on what is most relevant for the type of research question and causal hypothesis and on what knowledge is already available. 

In the process described in Figure 1, we first start from an observed phenomenon (stage 1), either in an individual or in a population. To arrive at a new hypothesis (stage 2), one needs to first gain a deeper understanding of the observed phenomenon in its context. This can be done by collecting evidence from qualitative research (e.g., ethnographic investigation) or case studies. Additionally, one must collect the existing knowledge, for example, through literature review, expert consultations or communication with other stakeholders. The hypothesis should include a disposition and its manifestation partners, which can then be tested with experimentation or further observation, or both. Testing with experimentation could be done in lab models or in populations (e.g., randomised controlled trials, N-of-1 trials). Testing with further observations can be done in population studies (e.g., case control, cohort studies) or by increased clinical alert (e.g., patient narrative). Once a disposition and its manifestation partners are established, or at least corroborated, causal inquiry is still not finished. Indeed, some of the most important information for broadening the causal understanding will come from observed cases of causal failure, in which the disposition does not manifest in the expected way. By collecting and investigating such unexpected outcomes and outlier cases, new manifestation partners and new dispositions might be detected. We have described some such cases above, in Section 4.

## 6. Concluding Remarks

From a dispositionalist perspective, establishing that an intervention causes an effect requires three forms of understanding, which should ultimately increase patient safety. First, we need to understand whether the intervention actually has an intrinsic disposition to produce the effect. Second, we need to understand which other dispositions or mutual manifestation partners played or could play a causal role in the process, either contributing to or counteracting the effect. Finally, we need to understand how these different dispositions interacted. This means that causal investigation must aim at theory development and mechanistic knowledge. 

In conclusion, we wish to anticipate one possible objection to our proposal. Such objection resonates an argument typically used in defence of the current evidence-based paradigm and can be summarised as follows. Is it feasible to prioritise the advance of theoretical knowledge, given the scarcity of resources for medical research, the huge costs and the amount of practical challenges that need to be addressed? Should medicine not be satisfied with finding solutions to problems, rather than using resources to investigate how and why these solutions work? This reasoning is built on the assumption that it is possible to make good medical research, aimed to answer the ‘whether’ and ‘how often’ questions, without the knowledge of ‘how’ and ‘why’. Such an assumption has been extensively addressed and challenged [2,10,13]. Theoretical understanding of medical observations and correlations is necessary to plan research, design experiments, interpret results, evaluate and weight experimental designs, and to use research findings in clinical decision making. A trade-off that de-prioritises theory building and understanding of phenomena, therefore, would ultimately result in an overall decrease in quality of medical research in general, and in an inefficient use of resources. Thus, the correct use of tools such as statistical significance, analysis of frequencies and statistical models ultimately depends on our theoretical understanding of human physiology, pathology, health and illness (see [13] for a detailed argument). We have here tried to show why such knowledge cannot be obtained by using one single methodological approach, but that different methods are useful for picking out different aspects of causality in medicine and public health.

## Figures and Tables

**Table 1:** A list of the research methods reviewed here, and an overview of their general purposes for the practice of causal inquiry.

Research Method	Purpose
**Experimentation with lab models**	To find causal mechanisms underlying observations
**Analysis of patient narrative**	To map detailed contextual information from the patient’s story that could contribute to explaining the patient’s current condition
**N-of-1 trials**	To test and compare the effects of different interventions within the same patient or context
**Case studies**	To analyse a medically interesting case or case series in sufficient detail, including relevant biographical data, social conditions, symptoms, diagnosis, management, and outcomes
**Case control and other retrospective studies**	To find a common cause in a population with a similar condition by comparing recorded medical data against a control
**Cohort studies and other prospective studies**	To find correlations between possible risk factors or susceptibility factors and health outcomes by observing a group of people over a period of time
**Randomised controlled trials**	To compare the outcome of one or more randomly assigned clinical intervention(s)

**Table 2:** Summary of the advantages and disadvantages of the different research methods for the purpose of uncovering causal dispositions and manifestation partners, with corresponding recommendations.

Research Method	Advantages for Detecting Dispositions	Disadvantages for Detecting Dispositions	Recommendations
Experimentation with lab models	Allow a study of causal processes in detail over time, including many dispositions and manifestation partners. Can inform causal mechanism by offering insight into interactions of manifestation partners under various controlled conditions.	The dispositions and manifestation partners studied in the lab are likely to interact differently in the context of application (the clinic).	Couple experimentation in lab models with clinical observations (especially where a disposition fails to manifest as expected) to gain understanding about a disposition and its various manifestation partners for a particular effect.
Analysis of patient narrative	Allow the detection of relevant dispositions that are uniquely combined in the patient. Facilitate the understanding of the causal story leading up to a condition, allowing a detection of various relevant dispositions and manifestation partners.	As with all in-depth analyses of one or few instances, it is difficult to narrow down which of the many elements in the patient narrative represent relevant dispositions for the illness or treatment.	Couple analysis of patient narratives with population studies and theoretical knowledge about what factors could influence the condition or treatment in general.
Case studies	Help understand how the dispositions at place interact with each other, by gaining a detailed overview of the context under scrutiny. Allow the description of rare or unexpected effects in a particular individual or context, suggesting previously unknown dispositions or mutual manifestation partners.	Plurality of perspectives is missed because information is organised into medical categories, filtered and interpreted in light of researchers’ knowledge, interest and research question. Information about the dispositions at place in the single case might be lost.	Include patient narratives for a qualitatively rich description of the case in order to include all the potentially important causal information. Couple cases studies with existing theoretical knowledge and experimentation in order to exclude irrelevant dispositions.
N of 1 trials	Help reveal how different dispositions manifest in interaction with one particular patient and their unique combination of dispositions and manifestation partners.	Establish only a correlation and not an intrinsic disposition.	Couple N of 1 trials with theoretical knowledge and high quality patient-based evidence in order to investigate whether the correlation might point to an intrinsic disposition.
Case control and other retrospective studies	Allow detection of rare dispositions through a detailed causal study of small samples, such as outlier cases or very rare conditions. Acknowledge the temporal aspect of causality, which in turn contribute to discover a causal mechanism.	Look only for common dispositions across different contexts and not for context-specific manifestation partners. As with all statistical studies in populations, these studies point at correlations which are not necessarily intrinsic dispositions.	Couple retrospective studies with experimentation in lab models and review existing theoretical knowledge to investigate whether the correlation might point to an intrinsic disposition.
Cohort studies and other prospective studies	Acknowledge the temporal aspect of causality. Can potentially address causal complexity and the interactions of different manifestation partners.	Difficult to predict how long a disposition will take to manifest at population level. When resources are limited, priority is given to large numbers of people over large numbers of factors monitored, hindering the potential for uncovering causal complexity.	Couple prospective studies with case studies, patient narratives and mechanistic evidence to investigate whether the correlation might point to an intrinsic disposition.
Randomised controlled trials	Well-suited to detect a difference in outcome at population level, which dispositions tend to do (but not always).	Identify all difference-makers irrespectively of whether they are causes or simply necessary conditions for the outcome. Not well-suited to identify multiple manifestation partners (causal complexity).	Couple RCTs with lab experimentation, case control studies and case studies to investigate causal interactions and manifestation partners.
